# Downregulation of the LncRNA MEG3 Promotes Osteogenic Differentiation of BMSCs and Bone Repairing by Activating Wnt/β-Catenin Signaling Pathway

**DOI:** 10.3390/jcm11020395

**Published:** 2022-01-13

**Authors:** Juan Liu, Xin Qi, Xiao-Hong Wang, Hong-Sheng Miao, Zi-Chao Xue, Le-Le Zhang, San-Hu Zhao, Liang-Hao Wu, Guo-Yi Gao, Mei-Qing Lou, Cheng-Qing Yi

**Affiliations:** 1 Department of Neurosurgery, Shanghai General Hospital, Shanghai Jiao Tong University School of Medicine, Shanghai 201620, China; pajingsen@126.com (J.L.); miaohongsheng1973@aliyun.com (H.-S.M.); zhaosanb@hotmail.com (S.-H.Z.); gao3@sina.com (G.-Y.G.); 2 Department of Orthopaedics, Shanghai Pudong Hospital, Fudan University Pudong Medical Center, No. 2800 Gongwei Road, Huinan Town, Pudong, Shanghai 201399, China; 912593451@qq.com (X.Q.); 1090061339@qq.com (L.-H.W.); 3 Department of Operating Room, Shanghai Pudong Hospital, Fudan University Pudong Medical Center, No. 2800 Gongwei Road, Huinan Town, Pudong, Shanghai 201399, China; hdandqx@163.com; 4 Department of Orthopaedics, Qingdao Municipal Hospital, Qingdao 266001, China; xzch_2230@163.com; 5 Department of Nursing, Shanghai Pudong Hospital, Fudan University Pudong Medical Center, No. 2800 Gongwei Road, Huinan Town, Pudong, Shanghai 201399, China; happy10lele@163.com

**Keywords:** long non-coding RNA, MEG3, BMSCs, skull defect, bone regeneration

## Abstract

Background: Previous studies have demonstrated that long non-coding RNA maternally expressed gene 3 (MEG3) emerged as a key regulator in development and tumorigenesis. This study aims to investigate the function and mechanism of MEG3 in osteogenic differentiation of bone marrow mesenchymal stem cells (BMSCs) and explores the use of MEG3 in skull defects bone repairing. Methods: Endogenous expression of MEG3 during BMSCs osteogenic differentiation was detected by quantitative real-time polymerase chain reaction (qPCR). MEG3 was knockdown in BMSCs by lentiviral transduction. The proliferation, osteogenic-related genes and proteins expression of MEG3 knockdown BMSCs were assessed by Cell Counting Kit-8 (CCK-8) assay, qPCR, alizarin red and alkaline phosphatase staining. Western blot was used to detect β-catenin expression in MEG3 knockdown BMSCs. Dickkopf 1 (DKK1) was used to block wnt/β-catenin pathway. The osteogenic-related genes and proteins expression of MEG3 knockdown BMSCs after wnt/β-catenin inhibition were assessed by qPCR, alizarin red and alkaline phosphatase staining. MEG3 knockdown BMSCs scaffold with PHMG were implanted in a critical-sized skull defects of rat model. Micro-computed tomography(micro-CT), hematoxylin and eosin staining and immunohistochemistry were performed to evaluate the bone repairing. Results: Endogenous expression of MEG3 was increased during osteogenic differentiation of BMSCs. Downregulation of MEG3 could promote osteogenic differentiation of BMSCs in vitro. Notably, a further mechanism study revealed that MEG3 knockdown could activate Wnt/β-catenin signaling pathway in BMSCs. Wnt/β-catenin inhibition would impair MEG3-induced osteogenic differentiation of BMSCs. By using poly (3-hydroxybutyrate-co-3-hydroxyhexanoate, PHBHHx)-mesoporous bioactive glass (PHMG) scaffold with MEG3 knockdown BMSCs, we found that downregulation of MEG3 in BMSCs could accelerate bone repairing in a critical-sized skull defects rat model. Conclusions: Our study reveals the important role of MEG3 during osteogenic differentiation and bone regeneration. Thus, MEG3 engineered BMSCs may be effective potential therapeutic targets for skull defects.

## 1. Introduction

In the clinic, skull defects caused by trauma, severe infection, tumor resection and decompressive craniectomy due to refractory high intracranial pressure are very common in neurosurgery. Although autologous bone transplantation, allogeneic bone transplantation, titanium mesh or polyether-ether-ketone (PEEK) cranioplasty were widely used in the clinical treatment of skull defects, complications such as donor site morbidity, bone resorption/loosening graft infection, implant exposure and high costs remain unresolved [1,2,3]. Bone marrow mesenchymal stem cells (BMSCs) retain their self-renewal capability and have the potential to differentiate into a variety of cell types; hence, they are widely used for tissue repairing. Multiple studies have demonstrated that combinations of biomaterials, growth factors or gene modified BMSCs-based tissue engineering are promising alternative approaches to facilitate bone regeneration and have acquired satisfying results in repairing critical-sized bone defects [4,5,6].

Maternally expressed gene 3 (MEG3) is a maternally expressed long non-coding RNA, and increasing evidence has revealed that MEG3 emerged as a key regulator in the process of development and tumorigenesis [7,8]. We have previously reported that downregulation of MEG3 could promote angiogenesis after ischemic brain injury [9]. However, the function of MEG3 in osteogenic differentiation of MSCs and bone regeneration remains largely unknown. Recent studies have revealed that MEG3 inhibits the osteogenic differentiation of periodontal ligament cells [10], human dental pulp stem cells [11] and BMSCs from postmenopausal osteoporosis [12]. Downregulated MEG3 promotes osteogenic differentiation of human dental follicle stem cells [13], while Zheng et al. have proved that downregulation of MEG3 suppresses osteogenic differentiation of human adipose-derived stem cells [14]. Upregulation of MEG3 promotes osteogenic differentiation of MSCs from multiple myeloma and pediatric aplastic anemia patients [15,16]. In addition, Liu et al. showed that MEG3 is upregulated in non-union bone fracture, and silencing MEG3 could accelerate tibia fraction healing in mice [17]. These contradictory findings indicate that dysregulation of MEG3 might play an important role in osteogenic differentiation of MSCs and bone remodeling.

Wnt/β-catenin signaling is a crucial pathway in the skeleton controlling osteoblast differentiation and bone formation [18]. With wnt/β-catenin pathway acting, wnt ligands bind to Frizzled and low-density lipoprotein receptor-related protein 5/6 (LRP5/6) receptors and induce stabilization of cytoplasmic β-catenin by inhibiting glycogen synthase knase 3 beta (GSK3β). β-catenin migrates into nuclei and forms a complex with transcriptional factor Tcf/Lef that transactivates target genes of the Wnt signaling pathway [19,20]. Previous studies have shown that disruption of β-catenin affects the osteogenic differentiation of BMSCs [21,22,23] and leads to extensive bone marrow adiposity and low bone mass [24]. Interestingly, crosstalk between MEG3 and Wnt/β-catenin pathways affected tumorigenesis [25,26], osteogenic differentiation [13,27] and bone formation [17], suggesting MEG3 is a regulator of Wnt signaling.

**Scheme 1:**
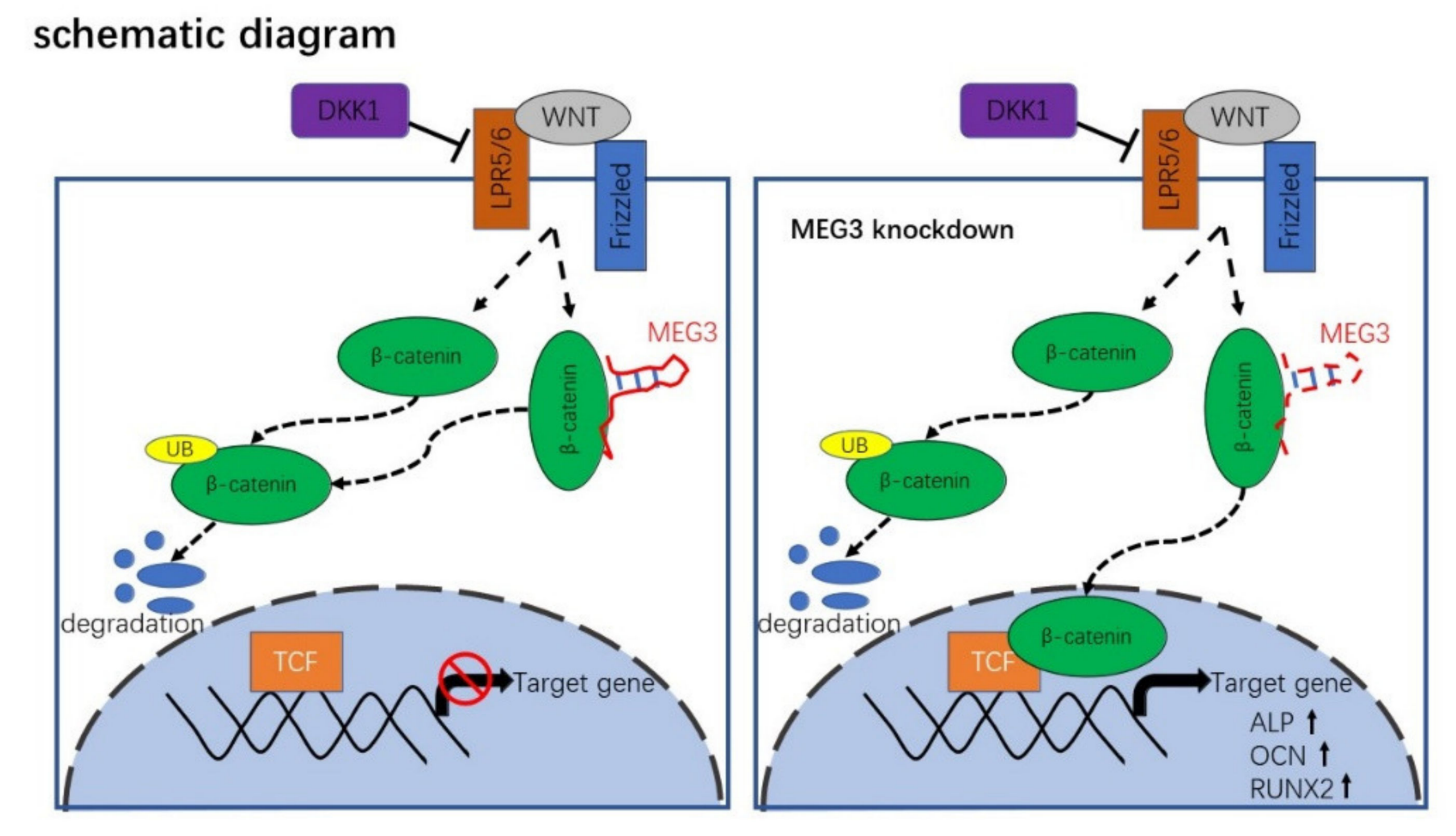
Scheme diagram of MEG3 knockdown promotes osteogenic differentiation of BMSCs by activating Wnt/β-catenin pathway.

In this study, we found the endogenous expression of MEG3 increased during the process of osteogenic differentiation. Based on previous studies and our results, we hypothesize that MEG3 may participate in osteogenic differentiation of BMSCs and bone regeneration. The function of MEG3 during osteogenic differentiation of BMSCs was demonstrated by assessing the expression levels of osteogenic genes, alkaline phosphatase activity and calcium deposition. Notably, a further mechanism study revealed that the pro-osteogenic effects of MEG3 may be partially attributed to wnt/β-catenin signaling pathway. A composite poly (3-hydroxybutyrate-co-3-hydroxyhexanoate, PHBHHx)-mesoporous bioactive glass (MBG) scaffold (PHMG), composed of biodegradable PHBHHx and MBG, was selected as the vehicle in this study, owing to the advantages of its good biocompatibility, favorable loading behavior of its mesoporous structure and so on. Our previous studies have shown that composite PHMG scaffolds have no cytotoxicity and improved cellular affinity; furthermore, PHMG possesses hierarchical mesoporous structure with a comparatively large specific surface area for cellular adhesion and spread [5]. PHMG scaffold adherent with MEG3 modified BMSCs was implanted in a critical-sized skull defect of rat model for 8 weeks. Bone regeneration was determined using micro-CT and histologic analyses. Our results show that downregulation of MEG3 could enhance osteogenic differentiation of BMSCs and promote bone repairing via Wnt/β-catenin signaling (Scheme 1).

## 2. Materials and Methods

### 2.1. Cells and Reagents

Human bone marrow stromal cells (hBMSCs) were obtained from four donors who gave their written informed consent. Briefly, marrow was extracted from the femoral midshaft and then suspended in minimum essential medium containing 10% fetal bovine serum (Hyclone; GE Healthcare, Little Chalfont, UK), 100 U/mL penicillin and 100 mg/L streptomycin. Subsequently, the non-adherent cells were discarded; the adherent cells converged to 80–90% confluence and were then replated as passage one (P1) cells. P3 cells were used for experiments. A density of 1 × 10^5^ cells/mL was used in the cellular tests. Recombinant DKK1 was purchased from PeproTech (Rocky Hill, NJ, USA). In accordance with a previous study, the applied concentration of DKK1 was 0.5 μg/mL [21,28].

### 2.2. Lentiviral Packaging and Cell Infection

Lentivirus knockdown MEG3 particles and lentiviral RFP particles were described as previously [9]. In brief, sh-Meg3, which specifically targeted MEG3, was inserted into pGLV10/U6/RFP/Puro vector (GenePharma, Shanghai, China). The lentiviral RFP particles were used as control group in this study. For infections, hBMSCs were incubated with lentiviral particles and polybrene (5 μg/mL) in growth medium. After 6 h, the infection medium was discarded. After 3 days, the cells were screened using puromycin (4 μg/mL; Sigma, Shanghai, China) and then passaged for use in subsequent experiments. The expression of MEG3 was quantified by quantitative real-time polymerase chain reaction (qPCR) and immunofluorescence.

### 2.3. Cell Counting Kit-8 (CCK-8)

To assess the effect of MEG3 downregulation on the proliferation of hBMSCs, the cells were seeded into a 96-well plate (5000/well) and allowed to adhere for 24 h. After 24 h, the medium was removed, and the cells were treated with 10% CCK-8 (Dojindo, Kumamoto, Japan) in 100 μL low-sugar Dulbecco’s modified Eagle’s medium (L-DMEM) without fetal bovine serum (FBS) for 2 h at 37 °C. Absorbance at 450 nm, which is directly proportional to cell proliferation, was measured using a microplate reader (ELX808; BioTek, Winooski, VT, USA).

### 2.4. Osteogenic Differentiation Protocol

BMSCs were cultured in growth medium (L-DMEM; 10% FBS (1495527; Gibco, Waltham, MA, USA) and 100 IU/mL penicillin/streptomycin) in 6- or 12-well cell culture plates (Corning, Shanghai, China), at a density of 3 × 10^4^/cm^2^, and incubated for 48 h at 37 °C under 5% CO_2_. The cells were subsequently cultured in osteogenic induction medium (L-DMEM with 10% FBS, 100 IU/mL penicillin/streptomycin, 100 nM dexamethasone, 0.2 mM ascorbic acid and 10 mM β-glycerophosphate). The cells were maintained by the addition of fresh osteogenic induction medium every 3 days.

### 2.5. Measurement of Alkaline Phosphatase (ALP) Activity

For the measurement of ALP activity, cells were lysed in radioimmunoprecipitation assay (RIPA) lysis buffer (Beyotime, Shanghai, China), and the lysate (10 μL) was incubated with 90 μL fresh solution containing p-nitrophenyl phosphate substrate at 37 °C for 30 min. The reaction was stopped by the addition of 0.5N NaOH (100 μL), and the absorbance was measured at 405 nm using a microplate reader (ELX808; BioTek). The total protein concentration was measured using a BCA protein assay kit (KeyGen BioTECH, Nanjing, China). The relative ALP activity is expressed as the percentage change in optical density (OD) per unit time per milligram protein: (OD/15 min/mg protein) × 100.

### 2.6. Alizarin Red Staining (ARS)

After the induction of osteogenic differentiation, mineral deposition was assessed by ARS (Cyagen Biosciences). Cells were fixed in 4% paraformaldehyde (Sangon Biotech, Shanghai, China) for 15 min at room temperature and then washed with distilled water. A 1% solution of alizarin red was added and incubated for 30 min at room temperature, followed by rinsing with distilled water. The solution was collected, and 200 μL were plated on 96-well plates, which were read at 560 nm using a microplate reader (ELX808; BioTek). The readings were normalized to the total protein concentration.

### 2.7. RNA Isolation and qPCR

Total cellular RNA was isolated using RNAiso reagent (Takara, Dalian, China) and quantified by measuring the absorbance at 260 nm (NanoDrop 2000; Thermo Fisher Scientific, Waltham, MA, USA). Total RNA (≤1000 ng) was reverse-transcribed into cDNA in a reaction volume of 20 μL using the Double-Strand cDNA Synthesis Kit (Takara, Dalian, China). One microliter of cDNA was used as the template for the qPCR reaction. All gene transcripts were quantified by qPCR using the Power SYBR Green PCR Master Mix (Takara) on the ABI StepOnePlus System (Applied Biosystems, Warrington, UK). The mRNAs of the target genes and GAPDH were quantified in separate tubes. All primers were synthesized by Sangon Biotech (Shanghai, China). The primer sequences used are shown in Table 1. The cycle conditions were as follows: 95 °C for 30 s and then 40 cycles of 95 °C for 5 s and 60 °C for 30 s. The relative target gene expression levels were calculated using the 2^−^^ΔΔ^^Ct^ method.

### 2.8. Western Blot Analysis

Cells were lysed in RIPA lysis buffer supplemented with a proteasome inhibitor (Beyotime). Total proteins were separated by 10% sodium dodecyl sulfate polyacrylamide gel electrophoresis and then transferred to a polyvinylidene fluoride membrane (Millipore, Shanghai, China). After blocking in 5% non-fat milk for 2 h, the membranes were incubated overnight at 4 °C with antibodies specific to β-actin (1:1000, Abcam Inc., Waltham, MA, USA) or β-catenin (1:1000, Cell Signaling Technology, Danvers, MA, USA). Horseradish peroxidase (HRP)-conjugated goat anti-rabbit IgG (1:1500; Cell Signaling Technology, USA) was applied as a secondary antibody for 2 h at room temperature. The immunoreactive bands were detected using an enhanced chemiluminescent detection reagent (Millipore, Shanghai, China). Signal intensity was measured using a Bio-Rad XRS chemiluminescence detection system (Bio-Rad, Hercules, CA, USA).

### 2.9. Cell Seeding

Prior to seeding cells, the prefabricated PHMG scaffolds were sterilized using gamma irradiation. Cell suspension (100 μL) was added to four groups at a density of 1 × 10^4^ cells/scaffold. After 4 h, 100 μL of culture medium was carefully added to the base of the culture plate until the scaffold was covered with sufficient culture medium.

### 2.10. In Vivo Evaluation in Animals

Animal experiments were approved by the Research Ethics Committee of the Shanghai General Hospital and performed in accordance with the Care and Use of Laboratory Animals protocols. Briefly, mature Sprague Dawley (SD) male rats (mean body weight 250–300 g) were provided with sterilized food and water and housed in a barrier facility with a 12-h light/dark cycle. These rats were randomly divided into three groups, each containing six rats: PHMG, PHMG + sh-Ctrl and PHMG + sh-MEG3 group. For the surgical procedure, as previously described, the animals were anesthetized by intraperitoneal injection of chloral hydrate (4%; 9 mL/kg body weight) and all operations were performed under sterile conditions. A 1.5-cm sagittal incision was made in the scalp and the calvarium was exposed by blunt dissection. Two critical-sized calvarial defects with a bilateral diameter of 5 mm were created using a dental trephine, and the scaffolds were then implanted into the defects. Following the operation, the animals received intramuscular antibiotic injections, were allowed free access to food and water and were monitored daily for potential complications. Eight weeks after the operation, the rats were killed by an overdose of anesthetics and their craniums were harvested and fixed in a 4% paraformaldehyde solution buffered with 0.1 M phosphate solution (pH 7.2) overnight before further analysis.

### 2.11. Micro-Computed Tomography (CT) Evaluation

All the harvested specimens were examined using the mCT-80 system to evaluate new bone formation within the defect region. Briefly, the undecalcified samples were scanned at a resolution of 18 μm and decalcified samples perfused with Microfil^®^ (Flowtech, Carver, MA, USA) were scanned at a resolution of 9 μm. After 3D reconstruction, the bone mineral density (BMD) and bone volume fraction (bone volume/total volume (BV/TV)) in the defect regions were used to calculate new bone formation using the auxiliary software of the mCT-80 system.

### 2.12. Histological and Immunohistochemical (IHC) Analysis

One part of calvarias was decalcified in 10% EDTA for 14 days, dehydrated with graded ethanol solutions, embedded in paraffin and sectioned at 5 μm at the central area of the defect. Sections were stained with hematoxylin and eosin (HE) to observe new bone formation.

The other part of each cranium was decalcified for approximately 2 weeks, dehydrated using a graded alcohol series, embedded in paraffin and sectioned into 5 μm sections. Osteocalcin (OCN) IHC was performed to evaluate osteogenesis in specimens. Briefly, deparaffinized sections were washed with PBS, treated with antigen retrieval and blocked with IgG for 30 min. OCN at a 1:200 dilution was incubated at 4 °C overnight. Then the biotinylated secondary antibody and ABC complex were applied and DAB substrate was used to stain the section. The sections were stained with hematoxylin and the results were analyzed with a light microscope.

### 2.13. Statistical Analysis

Statistical analysis was performed using SPSS 17.0 software (IBM, Armonk, NY, USA). All experiments were performed at least in triplicate, and the data are presented as means ± standard deviation. Statistical significance was determined using a two-tailed Student’s *t*-test when comparing two groups and one-way ANOVA followed by Bonferroni’s post hoc test when comparing more than two groups. *p* < 0.05 was considered to indicate statistical significance.

## 3. Results

### 3.1. Endogenous MEG3 Was Increased during Osteogenic Differentiation of BMSCs

**Figure 1:**
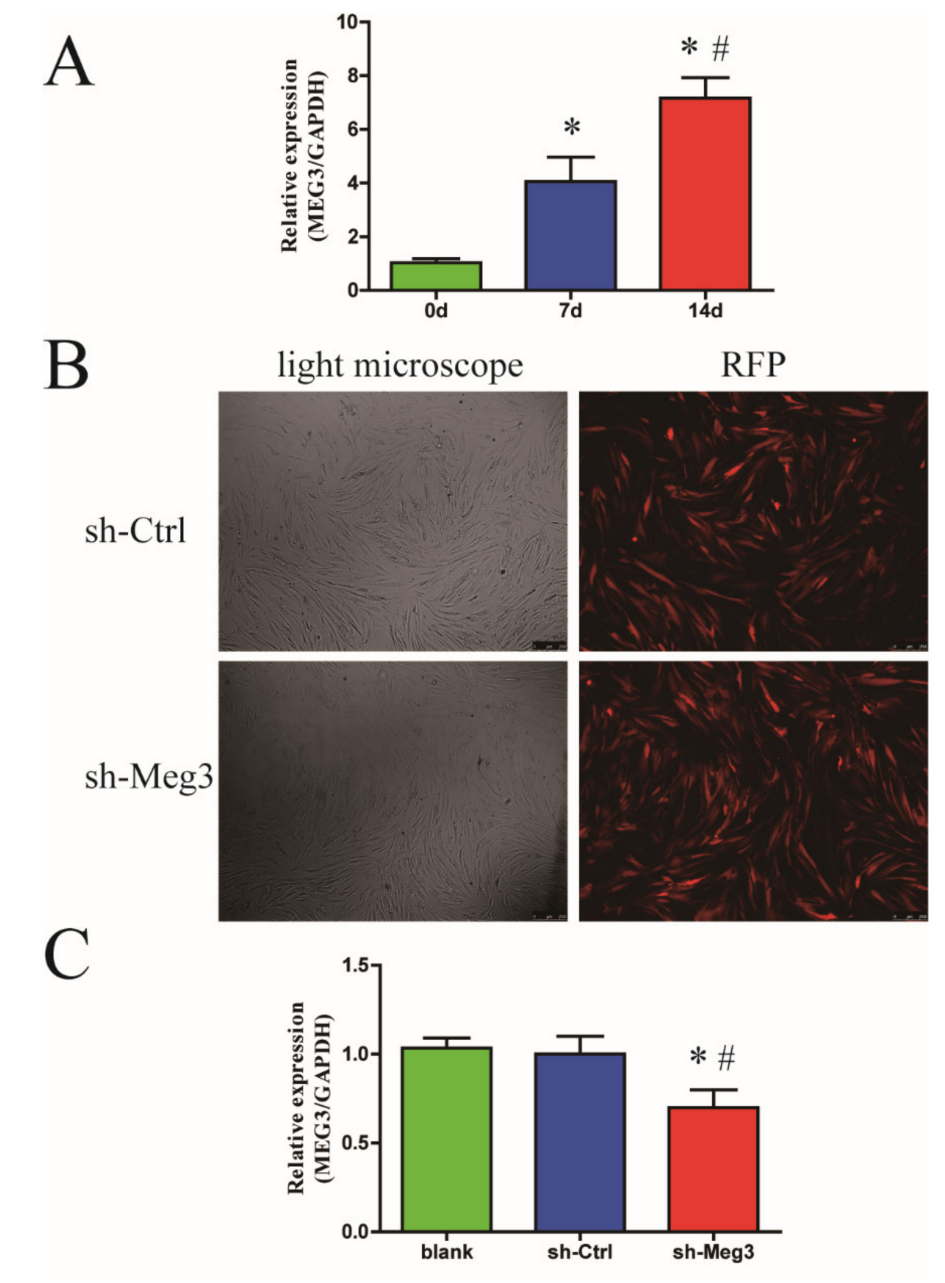
Expression of MEG3 during BMSCs osteogenic differentiation and MEG3 knockdown BMSCs. (**A**) Bar graphs show the expression levels of MEG3 in BMSCs at days 0, 7 and 14 of osteogenic differentiation. (**B**) BMSCs after lentiviral transfection were observed under an optical microscope and a fluorescence microscope. (**C**) The expression of MEG3 was determined by qPCR in lentivirus blank group, sh-Ctrl group and sh-Meg3 group (*n* = 3/group). * *p* < 0.05 versus the blank group. # *p* < 0.05 versus sh-Ctrl group.

To determine whether MEG3 was involved in osteogenic differentiation of BMSCs, we examined the expression MEG3 in BMSCs at day 0, 7 and 14 after osteoblastic induction. Compared with undifferentiated BMSCs, the expression of MEG3 was significantly increased at day 7 and 14 during the process of osteogenic differentiation (*p* < 0.05, Figure 1A).

To understand the role of MEG3 during osteogenic differentiation, we first performed MEG3 knockdown in the BMSCs using a lentivirus vector. Immunofluorescence showed that the RFP marker was stably expressed in the BMSCs (Figure 1B). PCR results showed the expression of MEG3 in the lenti-MEG3 treated BMSCs (sh-Meg3 group) were knockdown (0.7-fold) when compared with the mock treated BMSCs (blank group) and lenti-control treated BMSCs (sh-Ctrl group) (*p* < 0.05, Figure 1C). CCK8 results showed that no significant difference was detected in the cell proliferation rate between sh-Meg3 group and sh-Ctrl group at day 1, 3 and 7, which indicated MEG3 knockdown did not affect BMSCs proliferation (Figure S1).

### 3.2. MEG3 Knockdown Promotes Osteogenic Differentiation of BMSCs In Vitro

**Figure 2:**
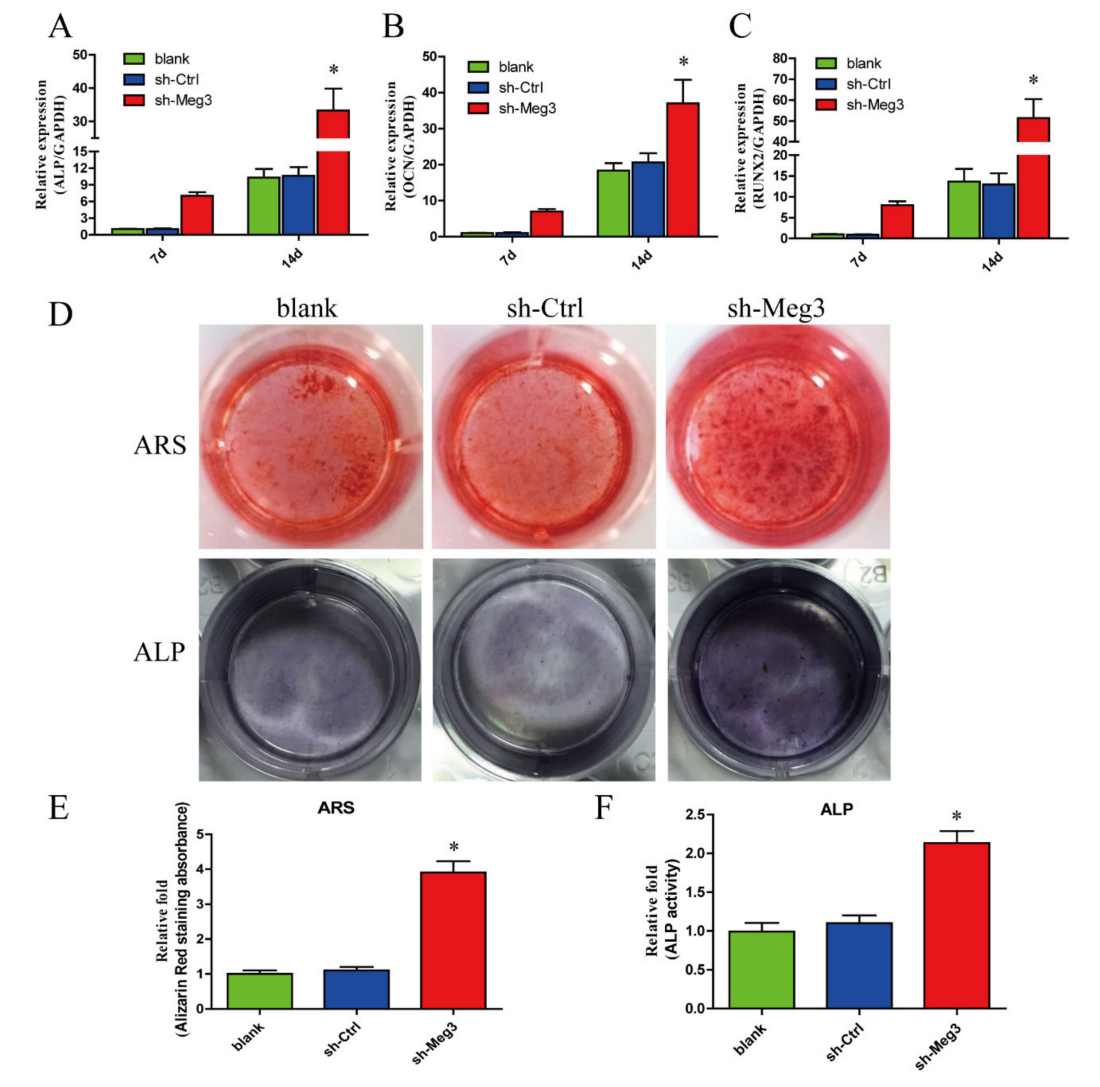
MEG3 knockdown promoted osteogenic differentiation of BMSCs. (**A**) Bar graph shows the qRT-PCR analysis of ALP expression at days 7 and 14 of osteogenic differentiation. (**B**) Bar graph shows the qRT-PCR analysis of OCN expression at days 7 and 14 of osteogenic differentiation. (**C**) Bar graph shows the qRT-PCR analysis of RUNX2 expression at days 7 and 14 of osteogenic differentiation. (**D**) ARS and ALP staining in the blank, sh-ctrl and sh-Meg3 groups at day 14 of osteogenic differentiation. (**E**) Alizarin red staining area in the blank, sh-ctrl and sh-Meg3 groups determined by measuring the absorbance at 560 nm. (**F**) ALP activity in the blank, sh-ctrl and sh-Meg3 groups at day 14 of osteogenic differentiation. Data are presented as mean ± SD. * *p* < 0.05 versus sh-Ctrl group (*n* = 3/group).

To further explore the function of MEG3 knockdown during osteogenic differentiation, the levels of osteo-specific genes, including ALP, runt-related transcription factor 2 (RUNX2) and osteocalcin (OCN) were detected by qPCR. qPCR analysis revealed that ALP, RUNX2 and OCN mRNA levels were significantly higher in sh-Meg3 group at day 7 and 14 than in blank group and sh-Ctrl group (*p* < 0.05, Figure 2A–C).

We evaluated ALP activity, an early marker of osteogenesis, at day 14 during osteogenic differentiation. Compared with the blank group and sh-Ctrl group, higher ALP activity was observed in sh-Meg3 group (*p* < 0.05, Figure 2D,F). Calcium deposits were also examined by ARS, and the staining areas were quantified by measuring the absorbance at 560 nm. More calcium deposits appeared in the sh-Meg3 group than in blank group and sh-Ctrl group at day 28 (*p* < 0.05, Figure 2D,E).

### 3.3. MEG3 Knockdown Activates Wnt/β-Catenin Signaling Pathway in BMSCs

**Figure 3:**
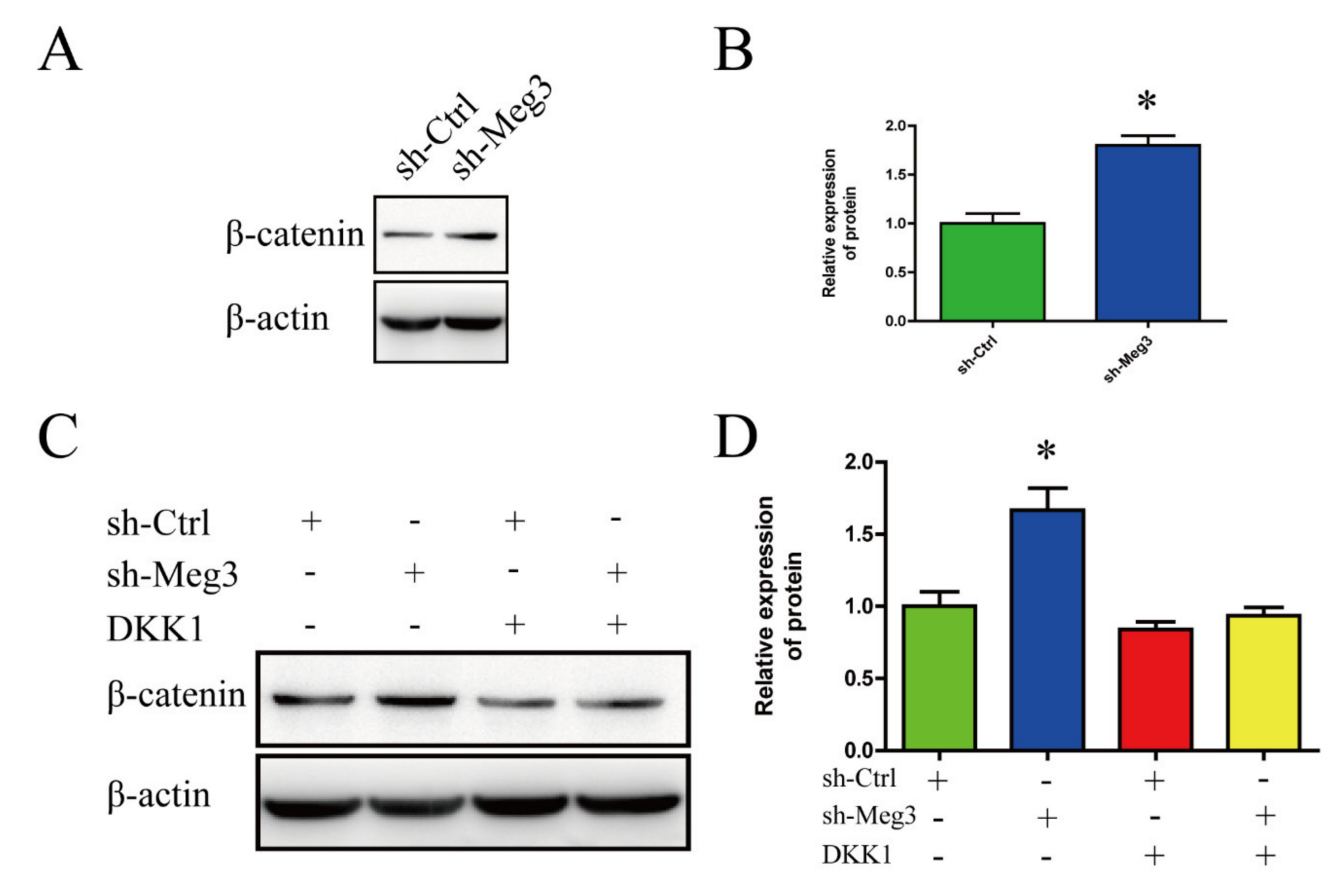
MEG3 regulates wnt/β-catenin signaling. (**A**,**B**) Representative immunoblot and bar graph show the relative expression level of β-catenin in sh-ctrl and sh-Meg3. (**C**) sh-Ctrl and sh-Meg3 BMSCs were treated with or without Wnt/β-catenin signaling inhibitor (DKK1). The expression of β-catenin in sh-ctrl, sh-ctrl + DKK1, sh-Meg3, and sh-Meg3 + DKK1 groups were determined by Western blot analysis. (**D**) Bar graph shows quantification expression of β-catenin in sh-ctrl, sh-ctrl + DKK1, sh-Meg3, and sh-Meg3 + DKK1 groups (n = 3/group). * *p* < 0.05 versus sh-ctrl.

Wnt/β-catenin signaling pathway plays an important role in the osteogenic differentiation of BMSCs. To gain insights into the mechanism by which MEG3 regulates osteogenic differentiation of BMSCs, the expression changes of β-catenin in BMSCs were performed. Western blot results showed that the protein level of β-catenin increased in the sh-Meg3 group when compared with sh-Ctrl group (*p* < 0.05, Figure 3A,B). DKK1 was previously reported as an inhibitor of Wnt/β-catenin pathway.

To verify the relevance of the Wnt/β-catenin pathway and MEG3 knockdown, we evaluated the expression of β-catenin between sh-Ctrl group and sh-Meg3 group treated with or without DKK1. Western blot results showed that MEG3 knockdown resulted in an increase of β-catenin in sh-Meg3 group compared to the sh-Ctrl group, and the upregulation of β-catenin could inhibit by DKK1 (*p* < 0.05, Figure 3C,D). Taken together, these data indicate that MEG3 activates the Wnt/β-catenin signaling pathway in BMSCs.

### 3.4. Wnt/β-Catenin Inhibition Impairs MEG3-Induced Osteogenic Differentiation of BMSCs

**Figure 4:**
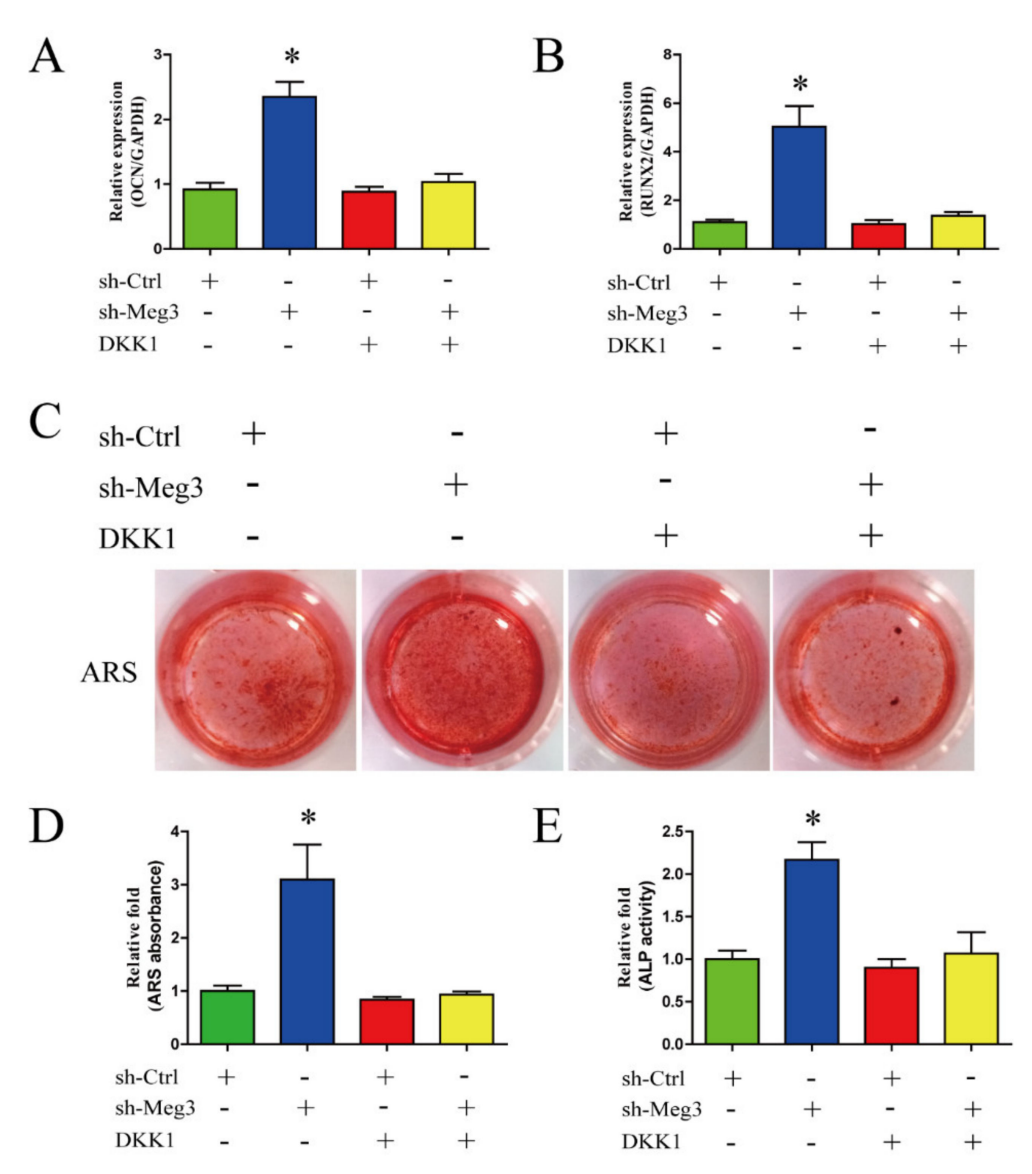
Wnt/β-catenin inhibition impairs MEG3-induced osteogenesis of BMSCs. (**A**) sh-Ctrl and sh-Meg3 BMSCs were treated with or without Wnt/β-catenin signaling inhibitor (DKK1). Bar graph shows the expression of OCN mRNA in sh-ctrl, sh-ctrl + DKK1, sh-Meg3 and sh-Meg3 + DKK1 groups. (**B**) Bar graph shows the expression of RUNX2 mRNA in sh-ctrl, sh-ctrl + DKK1, sh-Meg3 and sh-Meg3 + DKK1 groups. (**C**) Alizarin red staining in the sh-ctrl, sh-ctrl + DKK1, sh-Meg3 and sh-Meg3 + DKK1 groups at day 14 of osteogenic differentiation. (**D**) Alizarin red staining area in the sh-ctrl, sh-ctrl + DKK1, sh-Meg3 and sh-Meg3 + DKK1 groups determined by measuring the absorbance at 560 nm. (**E**) Bar graph shows expression of ALP in sh-ctrl, sh-ctrl + DKK1, sh-Meg3 and sh-Meg3 + DKK1 groups. Data are presented as mean ± SD. * *p* < 0.05 versus the sh-ctrl group (*n* = 3/group).

Next, we determined whether the osteogenesis effect of MEG3 knockdown on BMSCs was mediated via the Wnt/β-catenin pathway. After being treated with or without DKK1, osteo-specific genes of OCN and Runx2 were detected by qPCR in sh-Ctrl group and sh-Meg3 group. Compared with the sh-Ctrl group, silencing of MEG3 increased the expression of OCN and Runx2 in sh-Meg3 group, whereas the increasing of expression of OCN and Runx2 could blocked by DKK1 in sh-Meg3 group (*p* < 0.05, Figure 4A,B).

Moreover, there was also less matrix mineralization at day 28 of osteogenic differentiation in the sh-Meg3 group treated with DKK1than in sh-Meg3 group (Figure 4C,D). Blocking the Wnt/β-catenin pathway was able to reverse the osteogenesis activity induced by knockdown of MEG3 as indicated by ALP activity (*p* < 0.05, Figure 4E). These results indicate that the Wnt/β-catenin pathway could mediate the osteogenesis effect of MEG3.

### 3.5. MEG3 Knockdown Accelerated Bone Repairing in a Rat Critical-Sized Skull Defect

**Figure 5:**
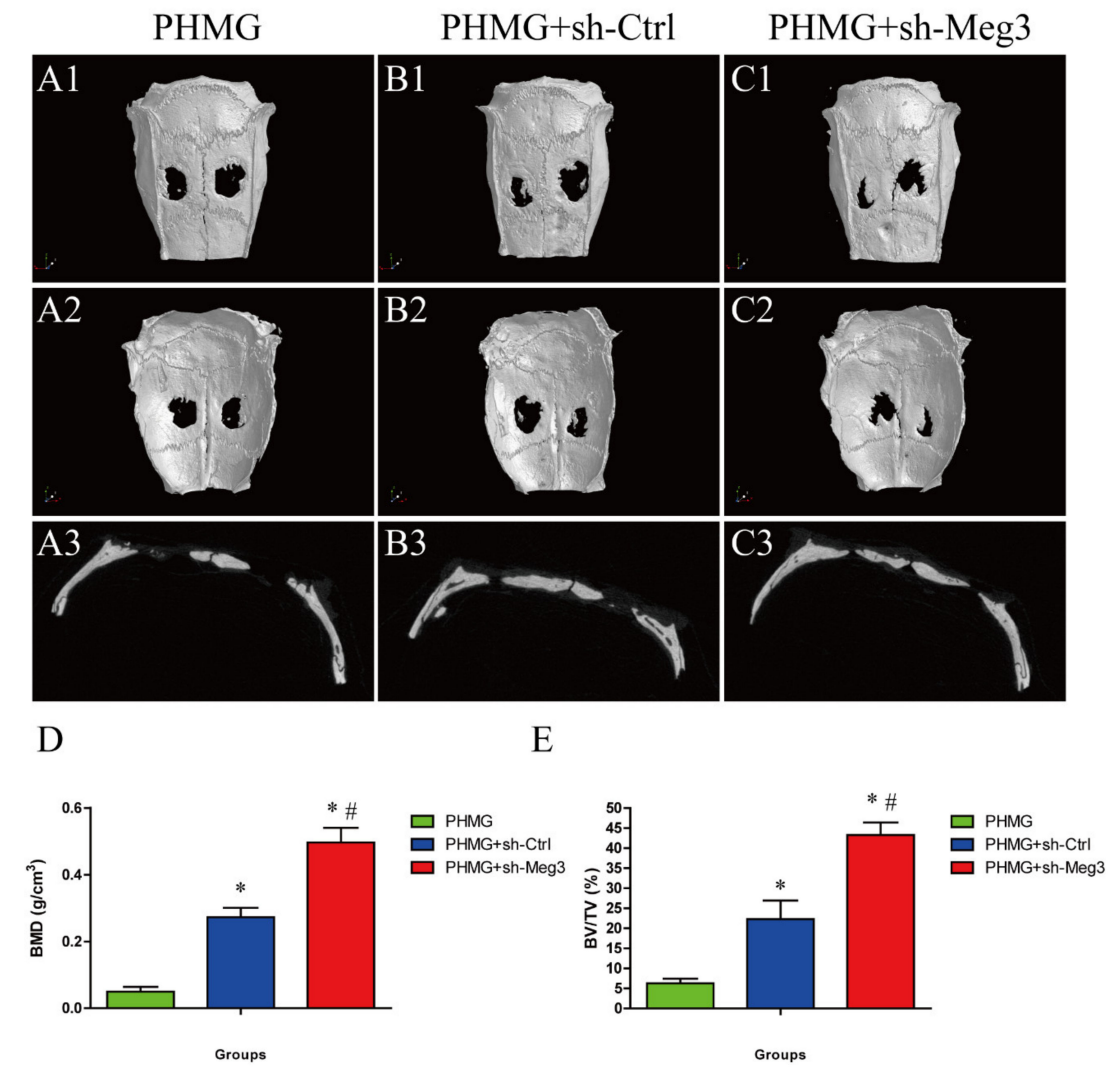
MEG3 knockdown in BMSCs promoted bone repairing in vivo. (**A1**–**C3**) Representative micro-CT images show the bone repairing in PHMG, PHMG + sh-Ctrl and PHMG +sh-Meg3 groups at 8 weeks. Bar graph shows quantitative analysis of micro-CT vessel volume in each group. Morphometric analysis of BMD (**D**) and BV/TV (**E**) as determined by micro-CT for each group at 8 weeks. * *p* < 0.05 versus the PHMG group, # *p* < 0.05 versus PHMG + sh-Ctrl group. (*n* = 6/group).

To verify the pro-osteogenesis effect of MEG3 in vivo, PHMG adherent with MEG3 knockdown BMSCs was implanted in a critical-sized skull defects of rat model. The 3D morphology and 2D slice images of the newly-formed calvarial bones of each group at week 8 are reconstructed by micro-CT (Figure 5(A1–C3)). As shown in the sagittal view, little bone growth was observed in the defect in the PHMG group (Figure 5(A3)); the PHMG + sh-Ctrl group showed increased new bone formation compared to the PHMG group (Figure 5(B3)). Compared with the PHMG group and the PHMG + sh-Ctrl group, newly-formed bone was apparently augmented in the PHMG + sh-Meg3 group, as the defect was almost completely filled with new calvarium, and the interfaces between the scaffold and bone tissues were connected (Figure 5(C3)). The local BMDs were markedly the highest at 0.497 ± 0.043 g/cm^3^, and there was a significant difference between the PHMG group and PHMG + sh-Meg3 groups (*p* < 0.05, Figure 5D). Moreover, BV/TV showed the same tendency as the BMD levels, there was a significant difference in the PHMG + sh-Meg3 group compared with the PHMG and PHMG + sh-Ctrl groups (*p* < 0.05, Figure 5E). These results indicate that PHMG scaffold with MEG3 knockdown BMSCs can synergistically improve bone regeneration compared to in vivo.

**Figure 6:**
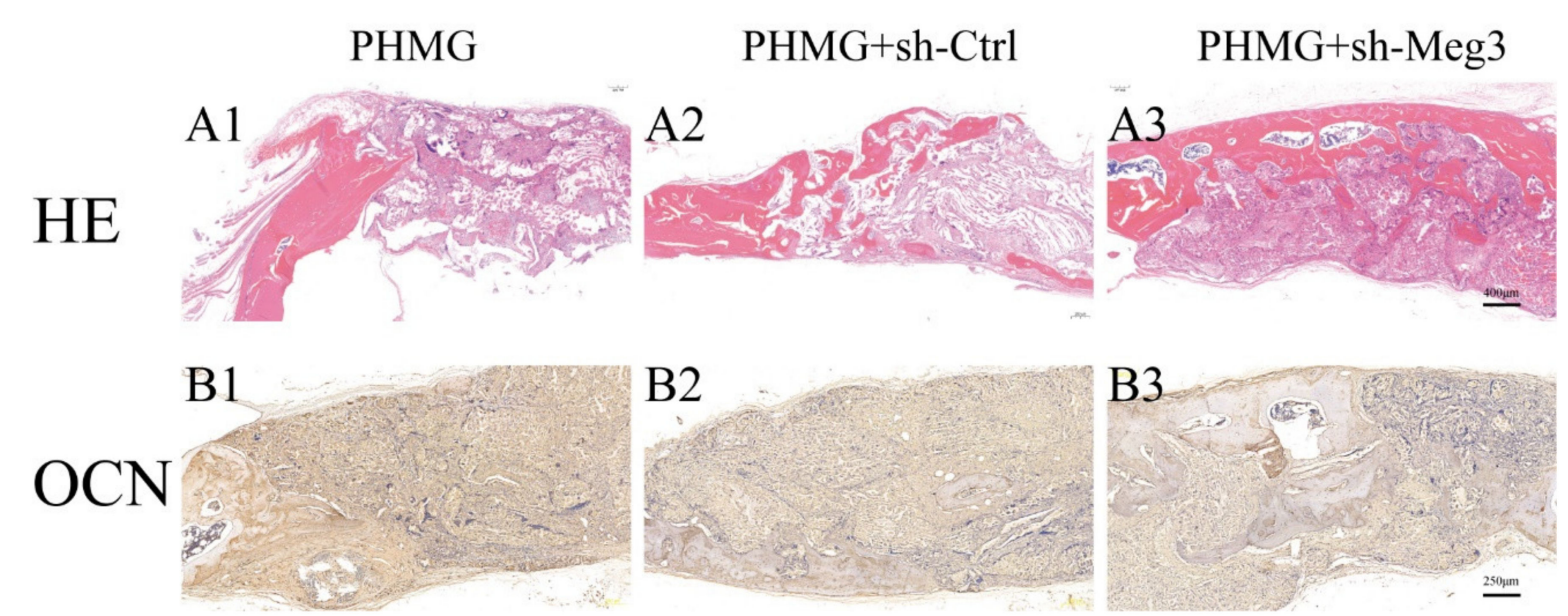
IHC analysis and OCN staining were used to detect osteogenesis of each group. (**A1**–**A3**) HE staining analysis, the red color represents new bone formation; (**B1**–**B3**) OCN staining analysis, the brown color represents positive staining of OCN. Bars = 400 μm in up panel and Bars = 250 μm in down panel.

HE staining clearly showed that barely any new bone formation was found in the PHMG group (Figure 6(A1)). Only a small amount of new bone formation was observed in the PHMG + sh-Ctrl group (Figure 6(A2)). Whereas in the PHMG + sh-Meg3 group, the ingrowth of new bone formation was evident in the central area of the defects as well as in the peripheral area near the pre-existing bones (Figure 6(A3)). The osteogenic marker OCN was also detected by IHC staining of decalcified craniums of each group. The results showed that there was less positive staining for OCN in the PHMG group (Figure 6(B1)). Positive brown staining for OCN was more apparent in the PHMG + sh-Ctrl (Figure 6(B2)) and PHMG + sh-Meg3 groups (Figure 6(B3)). HE and OCN IHC staining analysis of bone regeneration in calvarial defects indicated that Meg3 knockdown in BMSCs can increase bone regeneration.

## 4. Discussion

In this study, we found the endogenous expression of MEG3 was upregulated during osteogenesis in BMSCs. Furthermore, downregulation of MEG3 could promote the osteogenic differentiation of BMSCs. Moreover, we showed that decreasing the expression of MEG3 in BMSCs could markedly accelerate bone repairing with upregulated bone mineral density, bone volume and increased new bone generation in rat critical-sized skull defects model. Mechanistically, we found that silencing of MEG3 could promote osteogenic differentiation of BMSCs via the Wnt/β-catenin signaling pathway (Scheme 1). To our knowledge, this is the first report demonstrating that MEG3 enhances osteogenic differentiation of BMSCs, at least partly through activation of the Wnt/β-catenin signaling pathway.

BMSCs, combined with biomedical materials, hold great promise for regenerative medicine, especially for treating the unmet critical-sized bone defects in clinics. Therefore, effectively enhancing BMSCs osteogenic differentiation and BMSCs-mediated bone regeneration is crucial in bone tissue regeneration. Recent evidence has revealed that dysregulation of MEG3 is closely associated with bone or bone degenerative diseases. For example, Liu et al. found MEG3 is upregulated in non-union fracture bone [17] while MEG3 is downregulated in osteoarthritis [29,30]. In this study, we found the endogenous expression of MEG3 was upregulated during osteogenesis in BMSCs. RUNX2 is a master transcription factor involved in osteogenic differentiation. The expression of RUNX2 was significantly increased following the downregulation of MEG3. The levels of an early marker of osteogenic differentiation (ALP) and late markers of osteogenic differentiation (OCN) were also increased due to MEG3 depression. In agreement with our results, Li et al. also found the expression of serum lncRNA MEG3 was increased in fracture patients and intervention with MEG3 siRNA could obviously promote the proliferation and differentiation of osteoblast cell line MC3T3-E1 in vitro [27]. In addition, better bone healing was also observed when MEG3-modified BMSCs scaffold with PHMG used in a rat critical-sized skull defects model. Similarly, Liu et al. showed that silencing MEG3 could accelerate tibia fraction healing in mice [17]. However, some other studies have revealed the contradictory roles of MEG3 in osteogenic differentiation of BMSCs. Chen et al. found MEG3 may increase osteogenic differentiation of BMSCs in osteoporosis [31], and Zhuang et al. showed overexpression of MEG3 enhanced osteogenic differentiation of BMSCs from multiple myeloma patients [15]. Zheng et al. found MEG3 to be involved in the loss of odontogenic potential in dental mesenchymal cells, since MEG3 was significantly downregulated in cultured dental mesenchymal cells but was upregulated in odontogenic dental mesenchymal tissues [32]. Therefore, different expression of MEG3 in different cell types, tissues or diseases indicates MEG3 may have specific function under certain states.

Wnt/β-catenin signaling is an essential pathway in the osteogenic differentiation of MSCs and bone maintenance. Wnt signaling results in cellular accumulation of Wnt/β-catenin, followed by nuclear translocation of β-catenin and activation of target genes [18,33]. A previous study reported crosstalk between MEG3 and Wnt/β-catenin signaling in stem cells; that is, downregulated MEG3 promotes osteogenic differentiation of human dental follicle stem cells by epigenetically regulating Wnt pathway [13]. Gong et al. also found highly expressed MEG3 could weaken Wnt/β-catenin signaling in glioma [25]. MEG3 was reported to be located in the cytoplasm [13] and nucleus [17], suggesting that MEG3 could transfer between cytoplasm and nucleus. Studies have shown that lncRNAs such as MEG3 could act as competing endogenous RNA (ceRNA) or form RNA-DNA triplex structures, thereby regulating downstream genes and hence playing an important role in tumorigenesis and degenerative disease [7,11,26,34,35]. In this study, higher β-catenin accumulation was observed following downregulation of MEG3 during osteogenesis, suggesting that MEG3 downregulation activates β-catenin-mediated transcription. Furthermore, the increased osteogenesis of BMSCs by MEG3 downregulation was blocked by an inhibitor of Wnt/β-catenin. Since after the inhibition of canonical Wnt signaling pathway by DKK1, the osteogenic differentiation markers ALP, OCN and Runx2 were downregulated and less mineralized nodules were formed. Together, our data suggested that downregulated MEG3 might promote osteogenesis of BMSCs by partly activating the Wnt/β-catenin signaling pathway.

The present study has some limitations. First, although we indicated that downregulation of MEG3 mediates the Wnt/β-catenin signaling pathway to promote osteogenic differentiation of BMSCs, it is probably involved in the activation of other signaling pathways as well. We have previously demonstrated that MEG3 could interact with notch signaling to promote angiogenesis. Hence, other signaling pathways such as notch pathway could also play a role in MEG3 knockdown induced pro-osteogenic effect. Further work will be focused on these in our future research. Second, the mechanisms of MEG3 that interact with Wnt/β-catenin have not been clarified completely; research has shown that MEG3 could function as ceRNA or form RNA-DNA triplex structures to regulate gene expression and thus further studies are needed.

## 5. Conclusions

Based on our data, we found that downregulation of MEG3 could promote osteogenic differentiation of BMSCs and bone repairing, partly through activation of the Wnt/β-catenin signaling pathway. MEG3-modified BMSCs can be used as a novel promising strategy for skull defects.

## Figures and Tables

**Table 1:** Quantitative real-time polymerase chain reaction primer sequences.

Gene	Direction	Primer Sequence (5′-3′)
human GAPDH	Forward	ATCCCATCACCATCTTCC
	Reverse	GAGTCCTTCCACGATACCA
human ALP	Forward	GTTTTCTGTTCTGTAAGACGGG
	Reverse	GCCGTTAATTGACGTTCCGA
human RUNX-2	Forward	CCGAGCTACGAAATGCCTCT
	Reverse	GGACCGTCCACTGTCACTTT
human OCN	Forward	CCCCCTCTAGCCTAGGACC
	Reverse	ACCAGGTAATGCCAGTTTGC
human MEG3	Forward	GTTGAGCCTTCAGTGTCTGCAT
	Reverse	GCTTTGGAACCGCATCACA

## Supplementary Materials

The following are available online at https://www.mdpi.com/article/10.3390/jcm11020395/s1, Figure S1: The proliferation rate of BMSCs was not significantly affected by Meg3 downregulation.
