# Supplementary material for: Downregulation of the LncRNA MEG3 Promotes Osteogenic Differentiation of BMSCs and Bone Repairing by Activating Wnt/β-Catenin Signaling Pathway
Source: J Clin Med. 2022 Jan 13;11(2):395. doi: 10.3390/jcm11020395 (PMC8781453; doi:10.3390/jcm11020395)
Supplement: Supplementary file 1 [file jcm-11-00395-s001.zip › jcm-1492639-supplementary.pdf]

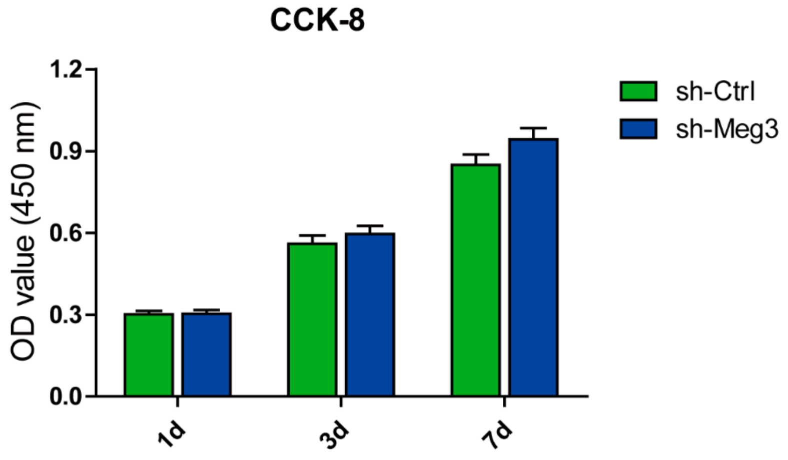

Figure S1. The proliferation rate of BMSCs was not significantly affected by Meg3 downregulation.
